# Radiosynthesis and validation of (±)-[^18^F]-3-fluoro-2-hydroxypropionate ([^18^F]-FLac) as a PET tracer of lactate to monitor MCT1-dependent lactate uptake in tumors

**DOI:** 10.18632/oncotarget.14705

**Published:** 2017-01-17

**Authors:** Vincent F. Van Hée, Daniel Labar, Gwenaël Dehon, Debora Grasso, Vincent Grégoire, Giulio G Muccioli, Raphaël Frédérick, Pierre Sonveaux

**Affiliations:** ^1^ Pole of Pharmacology, Institut de Recherche Expérimentale et Clinique (IREC), Université Catholique de Louvain (UCL), B-1200 Brussels, Belgium; ^2^ Pole of Molecular Imaging, Radiotherapy and Oncology, Institut de Recherche Expérimentale et Clinique (IREC), Université Catholique de Louvain (UCL), B-1200 Brussels, Belgium; ^3^ Louvain Drug Research Institute (LDRI), Université Catholique de Louvain (UCL), B-1200 Brussels, Belgium

**Keywords:** cancer metabolism, metabolic symbiosis, monocarboxylate transporter 1 (MCT1), MCT inhibitors, positron emission tomography (PET)

## Abstract

Cancers develop metabolic strategies to cope with their microenvironment often characterized by hypoxia, limited nutrient bioavailability and exposure to anticancer treatments. Among these strategies, the metabolic symbiosis based on the exchange of lactate between hypoxic/glycolytic cancer cells that convert glucose to lactate and oxidative cancer cells that preferentially use lactate as an oxidative fuel optimizes the bioavailability of glucose to hypoxic cancer cells. This metabolic cooperation has been described in various human cancers and can provide resistance to anti-angiogenic therapies. It depends on the expression and activity of monocarboxylate transporters (MCTs) at the cell membrane. MCT4 is the main facilitator of lactate export by glycolytic cancer cells, and MCT1 is adapted for lactate uptake by oxidative cancer cells. While MCT1 inhibitor AZD3965 is currently tested in phase I clinical trials and other inhibitors of lactate metabolism have been developed for anticancer therapy, predicting and monitoring a response to the inhibition of lactate uptake is still an unmet clinical need. Here, we report the synthesis, evaluation and in vivo validation of (±)-[^18^F]-3-fluoro-2-hydroxypropionate ([^18^F]-FLac) as a tracer of lactate for positron emission tomography. [^18^F]-FLac offers the possibility to monitor MCT1-dependent lactate uptake and inhibition in tumors *in vivo*.

## INTRODUCTION

Metabolic plasticity is a hallmark of cancer cells allowing them to optimally use existing resources for energy production and biosynthesis. Among possible fuels, lactate singles out as it is at the core of a metabolic cooperation between glycolytic cancer cells that produce lactate and oxidative cancer cells that use it [1]. This cooperation is of symbiotic nature: by delivering lactate to oxidative cancer cells that have a metabolic preference for lactate compared to glucose, glycolytic cancer cells facilitate glucose diffusion and use in the hypoxic/glycolytic cancer compartment [1–4]. Oxidative cancer cells, in turn, use lactate oxidation to promote autophagy, which has been linked to resistance to oxidative stress [5]. Together with other processes such as commensalism, autophagy and cannibalism, metabolic cooperativity represents an evolutionary solution for cell survival and proliferation in a metabolically altered environment [6].

At physiological pH, lactic acid (pKa 3.86) is fully dissociated in lactate and proton. Consequently, lactate swapping between glycolytic and oxidative cancer cells primarily depends on the expression and activity of lactate transporters of the monocarboxylate transporter (MCT) family that are located at the cell membrane [4, 7]. MCTs are passive transporters, among which MCT1 to MCT4 can transport lactate and are driven by the transmembrane gradient of lactate and protons. MCT4/SLC16A3, which has the lowest affinity for lactate (Km 22-28 mM) but a high turnover rate, is well adapted to facilitate the export of lactate and protons by glycolytic cancer cells [8–10]. This isoform is hypoxia-inducible (*SLC16A3* is a direct target gene of hypoxia-inducible factor-1 [HIF-1]) [11] and does not efficiently transport pyruvate (Km 153 mM) [4, 8, 12]. Comparatively, MCT1/SLC16A1 has a higher affinity for lactate (Km 3.5-10 mM) and can efficiently transport pyruvate (Km 1 mM) and ketone bodies [4, 12]. Although *SLC16A1* is not a direct HIF-1-target gene [11], experimental evidence showed that MCT1 expression can be induced by hypoxia in a HIF-1 dependent manner [13–16]. In cancers, MCT1 is preferentially expressed at the plasma membrane of oxidative cancer cells where it facilitates the uptake of lactate together with a proton, thereby alimenting the lactate oxidation pathway and supporting metabolic symbiosis [1]. MCT1 and MCT4 have further been involved in a commensalism behavior of oxidative cancer cells, whereby these cells mobilize and exploit lactate and ketone bodies produced by stromal cells [17–19]. Compared to MCT1 and MCT4, MCT2/SLC16A7 and MCT3/SLC16A8 are less often expressed in cancers [4].

Over the last 8 years, the existence of a metabolic symbiosis has been substantiated in different cancer types, indicating in general terms that this metabolic behavior is an important contributor to tumor progression. Evidence includes the preferential expression of MCT4 in the hypoxic/glycolytic cancer cell compartment and of MCT1 in well-oxygenated tumor areas, as well as the observation that ^13^C-labelled lactate can be converted into downstream metabolites of the lactate oxidative pathway (such as ^13^C-alanine) in tumors *in vivo* [20]. Overall, a metabolic symbiosis has been documented in a variety of human cancers, including head and neck, breast, lung, stomach, colon, bladder, prostate and cervix cancers, as well as gliomas [1, 3, 21–24]. This motivated the development and preclinical evaluation of several MCT inhibitors [25–29], among which AZD3965, initially developed as a mild immunosuppressor [30], is currently evaluated as an anticancer agent in Phase I clinical trials for patients with prostate cancer, gastric cancer or diffuse large B cell lymphoma (ClinicalTrials.gov NCT01791595). The related compound AR-C155858 is a selective MCT1 inhibitor that nevertheless also inhibits MCT2, but only when MCT2 is bound to ancillary protein basigin, whereas its preferred chaperon protein is embigin [31]. In this context, it is therefore of high interest that three independent studies recently assigned to metabolic symbiosis a primary responsibility for the induction of resistance to anti-angiogenic therapies [32–34], thus supporting the use of MCT inhibitors in combination with these treatments.

Although MCT1 inhibitors are being actively developed and AZD3965 recently entered into clinical trials for the treatment of cancer, there is currently no strategy allowing to measure lactate uptake and its inhibition in clinical settings. In this study, we report the original synthesis and preclinical validation of (±)-[^18^F]-3-fluoro-2-hydroxypropionate ([^18^F]-FLac) as a tracer of lactate uptake for positron emission tomography (PET). [^18^F]-FLac was generated in clinical settings and evaluated in the same cancer model that served for the discovery of the metabolic symbiosis of cancers.

## RESULTS

### (±)-[^18^F]-2-fluoropropionate ([^18^F]-FP) does not behave as a lactate tracer for PET imaging

**Figure 1:**
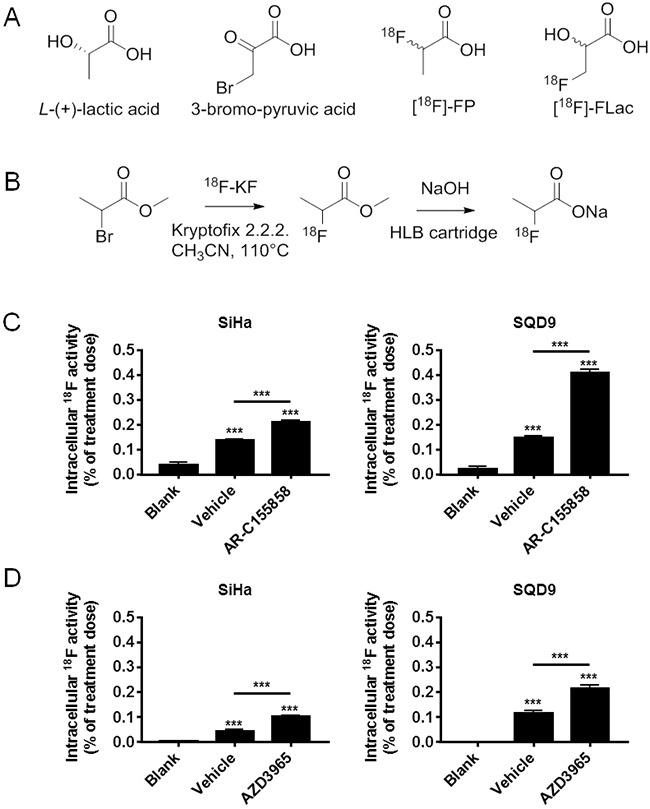
Oxidative human cancer cells do not trap (±)-[^18^F]-2-fluoropropionate in a MCT1-dependent manner **A**. Potential analogues of lactate considered for the development of a tracer of lactate uptake. **B**. Scheme for the radiosynthesis of (±)-[^18^F]-2-fluoropropionate. **C**. *In vitro* assay for the uptake of (±)-[^18^F]-2-fluoropropionate by oxidative SiHa (left panel) and oxidative SQD9 (right panel) cancer cells. Cells were pretreated during 1 h by vehicle or MCT1 inhibitor AR-C155858 (10 μM) in DMEM containing 10% of dialyzed FBS and 10 mM of *L*-lactate. This was followed by a 6 min incubation in a modified KREBS solution containing 10 mM of *L*-lactate, (±)-[^18^F]-2-fluoropropionate (45 μCi/mL) and 10 mM of *L*-lactate ± AR C155858 (10 μM) (*** p < 0.005; N = 2, n = 8). **D**. Same as C, but using MCT1 inhibitor AZD3965 (10 μM) instead of AR-C155858 (*** p < 0.001; N = 2, n = 8).

Because of chemical analogy with lactate (Figure 1A), we first considered using (±)-[^18^F]-2-fluoropropionate ([^18^F]-FP) as a potential tracer of lactate uptake in cancer. [^18^F]-FP was synthesized in a 30–40% radiochemical yield (Figure 1B). Before hydrolysis, (±)-[^18^F]-methyl 2-fluoropropionate was purified by semi-preparative HPLC to avoid contamination by 2-bromopropionate. [^18^F]-FP (45 μCi/mL) was provided to human SiHa cervix squamous cell carcinoma cells that were previously reported to be oxidative and to express the inward lactate transporter MCT1 [1, 35, 36, 37]. This cell line is the main model that served to identify metabolic symbiosis in 2008 [1]. The experiment was repeated on human SQD9 pharyngeal squamous cell carcinoma cells, another oxidative cancer cell line (see oximetry below). A 6 min incubation in the presence of 10 mM of *L*-lactate (carry on strategy) resulted in a cellular uptake of [^18^F]-FP that amounted only to ∼0.1% of the dose in the two cell lines (Figure 1C&1D). However, tracer uptake was not decreased but rather slightly increased when the cells were also treated by MCT1 inhibitors AR-C155858 (10 μM) (Figure 1C) and AZD3965 (10 μM) (Figure 1D). We therefore disqualified [^18^F]-FP as a tracer of lactate uptake.

### Original synthesis of (±)-[^18^F]-3-fluoro-2-hydroxypropionate ([^18^F]-FLac)

We next considered to develop (±)-[^18^F]-3-fluoro-2-hydroxypropionate ([^18^F]-FLac) as a tracer of lactate uptake. The choice was based on structural analogy with *L*-lactate and with 3-bromopyruvate, a well-known MCT1 substrate [38] (Figure 1A). At the time of our study, nonradioactive 3-fluoro-2-hydroxypropionate was not available commercially, but we verified experimentally that 3-fluoropyruvate could be converted to 3-fluoro-2-hydroxypropionate by lactate dehydrogenase (LDH), a bidirectional enzyme (Supplementary Figure 1A&1B). These data support the possibility that [^18^F]-FLac could be metabolized to [^18^F]-3-fluoropyruvate by LDH, *i.e*., along the oxidative pathway of lactate in oxidative cancer cells.

**Figure 2:**
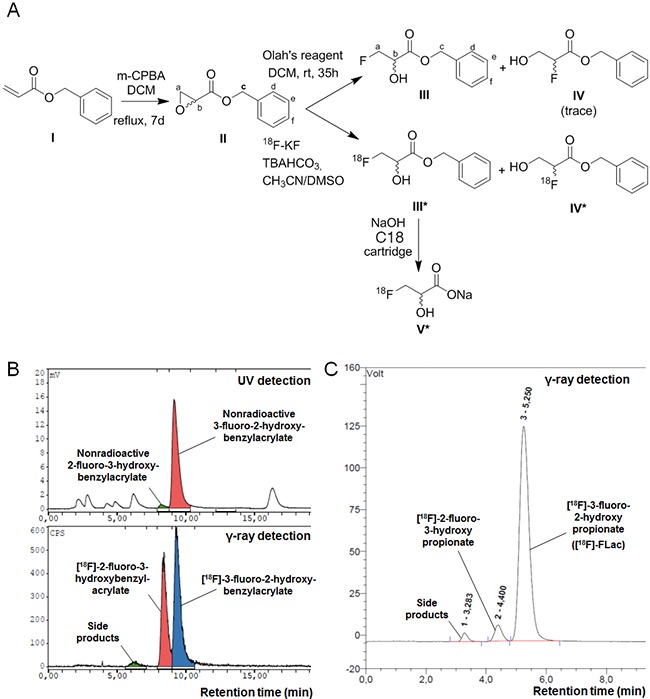
Synthesis of (±)-[^18^F]-3-fluoro-2-hydroxypropionate ([^18^F]-FLac) **A**. Scheme for the radiosynthesis of (±)-[^18^F]-3-fluoro-2-hydroxypropionate. **B**. Co-elution spectra of (±)-[^18^F]-benzyl 3-fluoro-2-hydroxypropionate and nonradioactive benzyl 3-fluoro-2-hydroxypropionate, and (±)-[^18^F]-2-fluoro-3-hydroxybenzylacrylate and nonradioactive 2-fluoro-3-hydroxybenzylacrylate on a Supelco Discovery C18 HPLC column equipped with UV and NaI γ-ray detectors. **C**. Elution spectrum of (±)-[^18^F]-3-fluoro-2-hydroxypropionate ([^18^F]-FLac) on a IonPac AS15 Dionex HPLC column equipped with a NaI γ-ray detector.

To our knowledge, [^18^F]-FLac has never been produced before. For the original synthesis of this potential tracer, we first synthesized benzyl oxirane-2-carboxylate (Compound II) by reacting 3-chloroperoxybenzoic acid with benzyl acrylate (Compound I) in DCM, as described in the Materials and Methods and shown in Figure 2A. The aromatic ring was introduced for UV detection. We obtained a yield of 53%, and benzyl oxirane-2-carboxylate was authenticated using ^1^H NMR (CDCl_3_ with 0.03% v/v TMS, 400 MHz): δ 7.38 (5.29H, m, H_d_, H_e_ and H_f_), 5.18-5.27 (2.7H, q, H_c_), 3.47-3.49 (1H, dd, H_b_), 2.94-3.01 (2.18H, qd, H_a_) (Figure 2A, Compound II).

[^18^F]-fluoride was generated on a medical isotope cyclotron. To optimize the opening of the epoxide ring and fluoro-hydroxy product stability in the reaction system, we used weakly basic conditions. [^18^F]-fluoride was trapped on a Chromafix 30-PS-HCO_3_ cartridge and was recovered (> 80%) by reverse order elution in a small volume of TBAHCO_3_. Benzyl oxirane-2-carboxylate was then reacted with [^18^F]-fluoride to yield 2 regioisomers: (±)-[^18^F]-benzyl 3-fluoro-2-hydroxypropionate (Compound III*) and (±)-[^18^F] benzyl 2-fluoro-3-hydroxypropionate (Compound IV*). The first radiofluorination reactions were conducted in DMSO at 120°C for 10 min (Method I), and about 90% of the [^18^F] radioactivity incorporated in organic molecules was related to both regioisomers (Compounds III* and IV*), with an about 1/1 ratio (Figure 2B). To improve regioselectivity, we conducted the radiofluorination reaction in 2-methyl 2-butanol (t-amyl-OH), a protic solvent (Method II). The regioselectivity of the epoxide opening improved up to more than 80% for (±)-[^18^F]-benzyl 3-fluoro-2-hydroxypropionate (Compound III*) (Supplementary Figure 2), and the global fluorination yield slightly increased (15-20%).

Compound III* and Compound IV* (NaI detector) co-eluted with nonradioactive benzyl 3-fluoro-2-hydroxypropionate (Compound III) and benzyl 2-fluoro-3-hydroxypropionate (Compound IV) (UV detector), respectively (Figure 2B). Benzyl 3-fluoro-2-hydroxypropionate (Compound III) was authenticated using ^1^H NMR (CDCl_3_ with 0.03% v/v TMS, 400 MHz): δ 7.35-7.39 (5.29H, m, H_d_, H_e_ and H_f_), 5.28 (1.85H, s, H_c_), 4.59 – 4.76 (1.94H, dqd, H_a_), 4.35 – 4.44 (0.92H, dquint, H_b_) (Figure 2A). Both (±)-[^18^F] regioisomers were isolated by semi-preparative HPLC. (±)-[^18^F]-Benzyl 3-fluoro-2-hydroxypropionate (Compound III*) was finally hydrolyzed in basic conditions to obtain [^18^F]-FLac (Compound V*, Figure 2A). Using a solid-phase extraction method with a C18 Sep-Pak cartridge and 0.5 N NaOH, hydrolysis was complete after 5 min. [^18^F]-FLac was characterized by analytical HPLC (IonPac AS15, Dionex, 14 mM of NaOH as eluent), with a retention time of 5.25 min (Figure 2C).

### Oxidative cancer cells take up and trap [^18^F]-FLac *in vitro*

**Figure 3:**
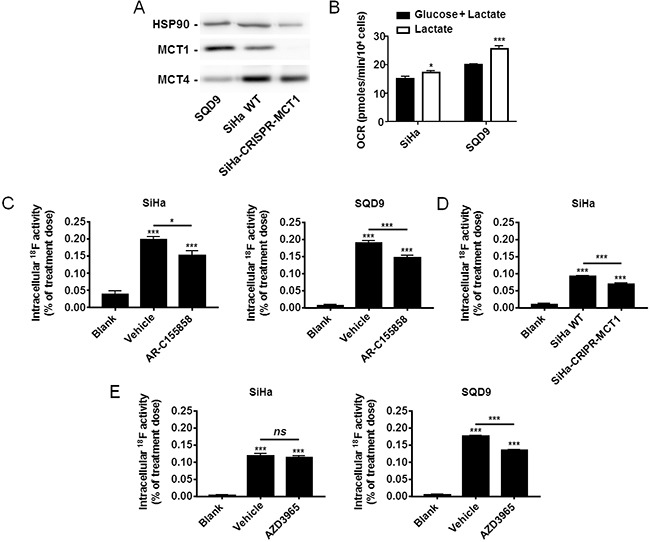
Oxidative human cancer cells trap [^18^F]-FLac in a MCT1-dependent manner **A**. Representative western blots showing MCT1 and MCT4 expression in SQD9, SiHa and SiHa-CRISPR-MCT1 human cancer cells. **B**. Oxygen consumption rate (OCR) of SiHa and SQD9 cells on a Seahorse bioanalyzer. Cells received either glucose (25 mM) + *L*-lactate (10 mM) (black bars) or only *L*-lactate (10 mM) (white bars) as oxidative fuels in DMEM containing 10% of dialyzed FBS (* p < 0.05, *** p < 0.005; n = 8). **C**. Cancer cells (or empty wells; blanks) were pretreated for 1 h with vehicle or AR-C155858 (10 μM) in DMEM containing 10% of dialyzed FBS and 10 mM of *L*-lactate, then incubated during 6 min in a modified KREBS solution containing 10 mM of *L*-lactate in the presence of (±)-[^18^F]-3-fluoro-2-hydroxypropionate ([^18^F]-FLac; 45 μCi/mL) with or without AR-C155858, washed, and intracellular ^18^F activity was measured using a Wiper Gold γ-counter (* p < 0.05, *** p < 0.001; N = 2, n = 8). **D**. As in C, except using SiHa-WT and SiHa-CRISPR-MCT1 cells without pretreatment (*** p < 0.001; N = 2, n = 8). **E**. As in C, but using AZD3965 (10 μM) instead of AR-C155858 (*ns*, p > 0.05; *** p < 0.001; N = 2, n = 8).

To evaluate [^18^F]-FLac as a potential tracer of lactate uptake by oxidative cancer cells, we selected SiHa and SQD9 cells. Indeed, the two cell lines did express MCT1 (Figure 3A), and oximetry on a Seahorse bioanalyzer confirmed that SQD9 were at least as oxidative as SiHa cells *in vitro* (Figure 3B) [1, 35, 36, 37]. As previously reported for SiHa [1], SQD9 cells could use lactate as an oxidative fuel in the absence of glucose (Figure 3B). *In vitro*, the cells took up and trapped [^18^F]-FLac 6 min after the delivery of 45 μCi/mL of the tracer (Figure 3C–3E). At this time, intracellular doses ranged from ∼0.1% for SiHa to ∼0.2% for SQD9 of the initial dose. This corresponded to an uptake of [^18^F]-FLac 1 to 2 times that of [^18^F]-FP. Pretreatment with MCT1 inhibitor AR-C155858 led to a significant decrease in [^18^F]-FLac uptake by both SiHa and SQD9 cells (Figure 3C). Inhibition of [^18^F]-FLac uptake by AR-C155858 was comparable to the reduction seen when MCT1 was deleted using a CRISPR/Cas9 strategy (Figure 3D). Comparatively, MCT1 inhibitor AZD3965 decreased [^18^F]-FLac uptake by SQD9 but not by SiHa cells at the time and dose tested (Figure 3E). Altogether, we considered that [^18^F]-FLac could potentially qualify as a tracer of lactate uptake by oxidative cancer cells.

### Validation of [^18^F]-FLac as a tracer of lactate uptake by tumors *in vivo*

**Figure 4:**
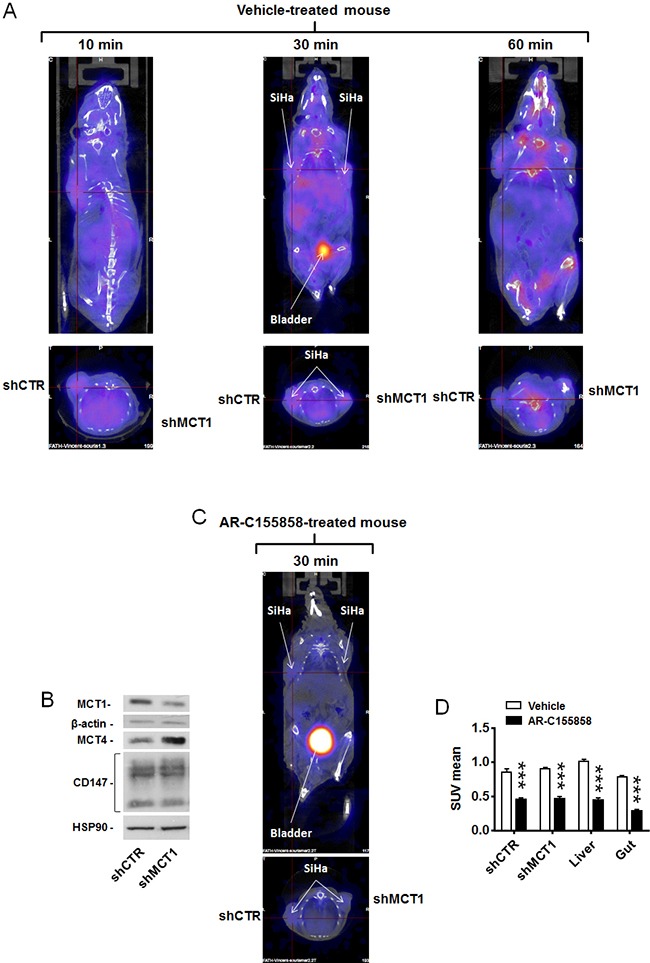
MCT1 inhibitor AR-C155858 blocks the *in vivo* uptake of [^18^F]-FLac by human SiHa tumors in mice Mice were bearing 2 SiHa tumors expressing a control shRNA (shCTR) or a shRNA against MCT1 (shMCT1). **A**. Representative images of vehicle-pretreated mice showing the physiological distribution of (±)-[^18^F]-3-fluoro-2-hydroxypropionate ([^18^F]-FLac) 10, 30 and 60 min after tail vein injection (215 μCi in 100 μL). Color scale is normalized for the injected dose and animal weight. **B**. Western blot showing MCT1, MCT4, CD147/basigin, β-actin and Hsp90 expression in SiHa cells infected with shCTR or shMCT1 (Representative of n = 3). **C**. Same as in A, except that mice were pretreated with AR-C155858 (5 mg/Kg IV 10 min before tracer injection). The representative image shows the exact same mouse as in A (30 min tracer image), assessed one day after. The bladder is indicated. **D**. Quantification of tracer uptake (30 min after tracer injection) based on PET scan images. A same group of mice was treated with vehicle (white bars) on one day and, on the day after, with AR-C155858 (5 mg/Kg IV 10 min before tracer injection) (black bars) (*** p < 0.001; N = 2, n = 6-7).

To validate [^18^F]-FLac as a tracer of lactate uptake by tumors, we used mice bearing 2 SiHa tumors that expressed shCTR in one flank and shMCT1 in the other flank. Tumor volumes measured on CT images 36 days after tumor implantation showed a significant difference between shCTR and shMCT1 tumors (Supplementary Figure 3). As both tumors were of sufficient size for proper imaging and no difference in SUV was observed, a partial volume effect was excluded. A same group of mice was used for vehicle treatment on one day and for treatment with MCT1 inhibitor AR-C155858 the day after. [^18^F]-FLac was administered intravenously at a dose of 150-250 μCi. With respect to vehicle treatment, PET/CT images revealed that tracer distribution was time-dependent, with best tumor contrast 30 min after tracer injection (Figure 4A and Supplementary Video 1). Other organs known to express MCTs and to take up lactate, such as the gut and the liver [7], were also labeled. Thirty minutes after tracer injection, there was no detectable bone labeling that could have indicated defluorination. Of note, 60 min after tracer injection, spinal and joint labeling was detected, indicating that some defluorination had possibly occurred (Figure 4A, right panel). At 30 min, there was no apparent discrimination of SiHa-shCTR and SiHa-shMCT1 by the tracer. This lack of difference was associated to an overexpression of MCT4 upon MCT1 silencing that we detected by western blotting (Figure 4B). On the day after, we used the same mice to evaluate the ability of [^18^F]-FLac to detect an acute pharmacological inhibition of MCT1, thus recapitulating at best a clinical treatment. One day after data acquisition for vehicle treatment, the mice were treated with MCT1 inhibitor AR-C155858 (5 mg/Kg) [31] administered intravenously 10 min before a PET/CT scan. Images acquired 30 min after [^18^F]-FLac delivery revealed that MCT1 inhibition by AR-C155858 induced a highly significant decrease in tracer uptake in the tumors, liver and gut (Figure 4C&4D and Supplementary Video 1). The bladder, which was already apparent on vehicle treatment images (Figure 4A, 30 min), was much more positively marked after systemic MCT1 inhibition (Figure 4C, 30 min), indicating that urine is the preferred route for [^18^F]-FLac clearance. Accordingly, kidneys, that preferentially express MCT2 for lactate clearance, were labelled after treatment. Note that, for comparison, PET/CT scans of the same mouse are shown in Figure 4A, Figure 4C and in Supplementary Video 1 at the 30 min acquisition time, with a color scale normalized for the injected dose.

**Figure 5:**
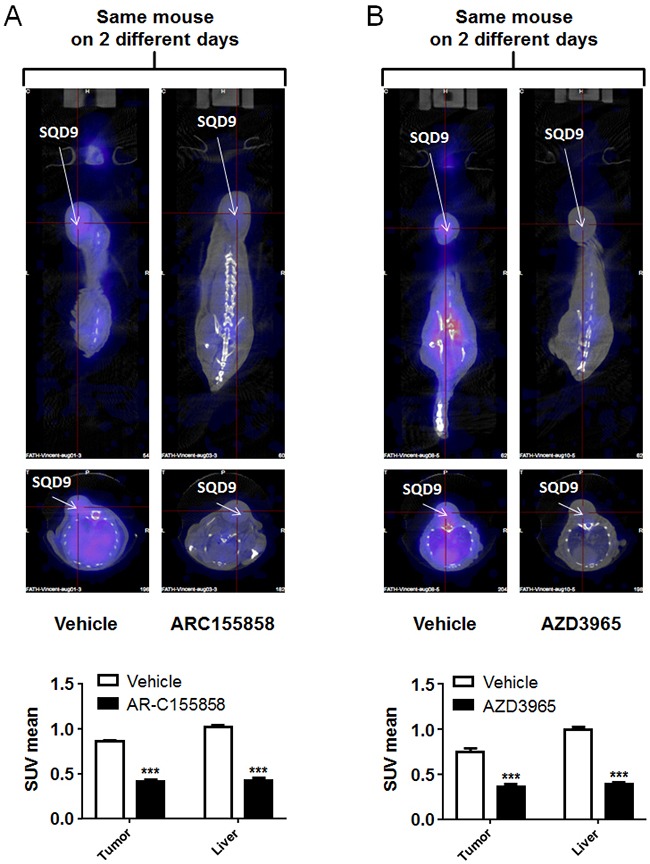
MCT1 inhibitors AR-C155858 and AZD3965 block the *in vivo* uptake of [^18^F]-FLac by human SQD9 tumors in mice Mice were bearing a single SQD9 tumor. **A**. Top left panel: representative PET image of a mouse that was pretreated for 10 min with vehicle, then imaged 30 min after a tail vein injection of (±)-[^18^F]-3-fluoro-2-hydroxypropionate ([^18^F]-FLac) (215 μCi in 100 μL). Top right panel: two days after, representative image of the exact same mouse that was pretreated for 10 min with AZD3965 (5 mg/Kg), then imaged 30 min after a tail vein injection of [^18^F]-FLac (215 μCi in 100 μL). Color scale is normalized for the injected dose and animal weight. The bottom graph shows quantification of tracer uptake based on PET scan images, where white bars correspond to vehicle treatment and black bars to AR-C155858 treatment (*** p < 0.001: N = 1, n = 6). **B**. Top left panel: representative PET image of a mouse that was pretreated for 10 min with vehicle, then imaged 30 min after a tail vein injection of [^18^F]-FLac (215 μCi in 100 μL). Top right panel: two days after, representative image of the exact same mouse that was pretreated for 10 min with AZD3965 (5 mg/Kg), then imaged 30 min after a tail vein injection of [^18^F]-FLac (215 μCi in 100 μL). Color scale is normalized for the injected dose and animal weight. The bottom graph shows quantification of tracer uptake based on PET scan images, where white bars correspond to vehicle treatment and black bars to AZD3965 treatment (*** p < 0.001: N = 1, n = 6).

Similar data were obtained when treating mice bearing a single wild-type SQD9 tumor with vehicle and MCT1 inhibitor AR-C155858 (Figure 5A and Supplementary Video 2; pictures of a same mouse imaged on different days are shown) and AZD3965 (Figure 5B and Supplementary Video 3; pictures of a same mouse imaged on different days are shown), thus confirming that [^18^F]-FLac can be used to image MCT1-dependent lactate uptake inhibition *in vivo*.

## DISCUSSION

This study aimed to identify and validate a tracer of lactate for PET imaging of tumors. We report that (±)-[^18^F]-3-fluoro-2-hydroxypropionate ([^18^F]-FLac) fulfills the necessary requirements, whereas (±)-[^18^F]-2-fluoropropionate does not. Indeed, we provide evidence that [^18^F]-FLac is actively taken up and retained by oxidative cancer cells that consume lactate *in vitro*, and accumulates in tumors and tissues known to consume lactate *in vivo*, which is efficiently prevented by the pharmacological inhibition of inward lactate transporter MCT1.

The vast majority of solid tumors comprise areas with limited oxygen and metabolite bioavailability surrounded by well perfused and oxygenated areas. Their temporal distribution varies [39] and is influenced by therapy [40]. This peculiar organization imposes a metabolic pressure on cancer cells that, in many cancer types [1, 3, 21–24], establish a metabolic symbiosis based on the exchange of lactate between glycolytic cancer cells that produce lactate and oxidative cancer cells that use it. The metabolic reward of lactate recycling is improved glucose availability for glycolytic cancer cells and increased autophagy for oxidative cells [5]. Consequently, the therapeutic strategy consisting in interfering with lactate exchanges and use in cancer is particularly attractive. While MCT1 inhibitor AZD3965 is currently evaluated as an anticancer agent in Phase I clinical trials (ClinicalTrials.gov NCT01791595) and other agents are in the pipeline [4, 25, 26], two burning questions are still unanswered: (*i*) is it possible to stratify patients that would benefit from such treatments, and (*ii*) is it possible to evidence early a therapeutic response to these agents?

Analyses of tumor biopsies are confronted to two major problems. First, MCT isoforms are generally segregated, with hypoxia-inducible MCT4 being preferentially expressed in hypoxic/glycolytic areas and MCT1 in oxygenated areas where cancer cells are equipped for lactate oxidation [1, 4, 22]. Thus, the presence of the therapeutic target in a given biopsy is influenced by sampling localization. A second limitation is that MCTs are passive transporters driven by the gradient of lactate and protons across the plasma membrane [7, 12]. Thus, even if detected in a biopsy, their presence provides no information about their activity. In addition, the metabolic use of lactate depends on intact and operational oxidative phosphorylation [1, 41], which cannot be easily assessed in clinical samples. This motivated us to develop a lactate analogue tracer for PET imaging.

Our rationale for the design of the tracer was based on the chemical structures of known MCT1 substrates, *i.e*., lactate and 3-bromopyruvate [38]. We initially investigated the synthesis of [^18^F]-FP because we expected that the bioisosteric replacement of the 2-hydroxyl function in lactate by a fluorine atom in the tracer would generate a compound that would still be a substrate of MCT1. [^18^F]-FP synthesis had already been described [42], and we obtained this compound in very good radiochemical yield following a two-steps strategy involving the substitution of the bromine of the methyl 2-bromopropionate by [^18^F]-fluoride anion, followed by saponification of the methyl ester. However, the biological evaluation of [^18^F]-FP showed that, although this compound was taken up by MCT1-expressing oxidative cells, it did not accumulate in a MCT1-dependent manner (Figure 1C–1D). [^18^F]-FP is indeed described as a tracer of lipid precursor acetate, not lactate [42]. Thus, the observation that [^18^F]-FP incorporation increased upon MCT1 inhibition probably reflects increased lipid uptake by oxidative cancer cells when lactate is not available as an oxidative fuel.

Having disqualified [^18^F]-FP as a lactate tracer, we aimed to investigate [^18^F]-FLac. This compound seemed very promising because it involved the bioisosteric replacement of an hydrogen atom in the 3-position of lactate with a fluorine, thus leaving the 2-hydroxyl function still available for a proper recognition by MCT1 and LDH, on the contrary to that of [^18^F]-FP. However, although the replacement of a hydrogen atom with a fluorine is usually reported to afford bioisosteric compounds, its introduction in the 3-position of lactate was very challenging because, to our knowledge, this radiolabeled compound has never been reported before. Its synthesis involved the opening of an epoxide precursor with fluorine, which is known to be favored at the least substituted carbon in the presence of an electron-donating group at the epoxide α-position [43, 44], which had never been investigated previously in the presence of an electron-attracting group at this α-position (in our case, carboxylate). The high electronegativity of the fluorine atom could have further impacted the electronic surrounding of the molecule, thus affecting its proper recognition by MCT1 and LDH. With an original radiosynthesis protocol on a clinical setup, we nevertheless succeeded to produce [^18^F]-FLac in a good radiochemical yield and with a good regioselectivity in favor of the targeted (±)-[^18^F]-3-fluoro-2-hydroxypropionate *vs*. (±)-[^18^F]-2-fluoro-3-hydroxypropionate.

[^18^F]-FLac accumulated in MCT1-expressing oxidative cancer cells similarly to [^18^F]-FP (Compare Figure 3C-3E and Figure 1C-1D). However, [^18^F]-FLac uptake was decreased by the pharmacological inhibition and genetic deletion of MCT1. We also provide evidence that the compound can be metabolized to [^18^F]-3-fluoropyruvate by LDH, a bidirectional enzyme (Supplementary Figure 1). A general characteristic of clinically used PET tracers is precisely their ability to be metabolized intracellularly for cell trapping [45]. For example, thymidine analogue tracer 3′-[^18^F]-fluoro-3′-deoxythymidine ([^18^F]-FLT), a marker of cell proliferation, is incorporated in DNA following processing by the thymidine nucleotide salvage pathway [46]. In another example, after its uptake by passive glucose transporters, glucose analogue tracer 2-deoxy-2-[^18^F]-fluoro-*D*-glucose ([^18^F]-FDG) is converted to [^18^F]-FDG-6-phosphate in order to label cancer cells characterized by a high uptake rate of glucose and high hexokinase activity [47]. By analogy with [^18^F]-FDG, we propose that, after its uptake by passive MCTs, [^18^F]-FLac is processed by the oxidative pathway of lactate, which would explain why MCT1-expressing oxidative cancer cells do accumulate the tracer (Figure 3). This possibility, however, remains to be investigated.

[^18^F]-FLac labelled SiHa and SQD9 tumors *in vivo*, as well as the gut and liver of mice, *i.e*., tissues that are well known to actively take up lactate [7]. However, the tracer was not capable to discriminate between shCTL and shMCT1 tumors, which we attribute to increased MCT4 expression upon MCT1 silencing (Figure 4). MCT4 overexpression was previously shown to functionally compensate for loss of MCT1 expression in glycolytic cancer cells *in vitro* [48, 49]. However, in our *in vivo* model, MCT4 activity would only partially rescue lactate uptake in the absence of MCT1, as the extracellular concentration of lactate is expected to be below the Km of MCT4 (Km lactate = 22-28 mM). Because[^18^F]-FLac uptake was not modified in shCTL *versus* shMCT1 tumors, other transporters could be involved as well. MCT1 and MCT4 share CD147/basigin as a chaperon protein for stable expression of the transporters at the cell membrane [50]. Increased basigin availability for interaction with MCT4 probably explains higher MCT4 expression in our model. We therefore considered acute pharmacological inhibition as a situation closer to clinical reality. Strikingly, [^18^F]-FLac allowed to unambiguously identify animals acutely exposed to MCT1 inhibitors AR-C155858 [31] and AZD3965 [29]. Treated animals were characterized by a complete loss of [^18^F]-FLac incorporation in tumors, gut and liver, whereas the kidneys (which preferentially express MCT2 for lactate clearance [7]) and the bladder were strongly labeled (Figure 4C–4D, Figure 5A–5B, Supplementary Video 1, Supplementary Video 2 and Supplementary Video 3). This set of data clearly indicates that [^18^F]-FLac can detect lactate flux inhibition *in vivo*, and it did so as early as 40 min after the intravenous delivery of AR-C155858, at clinically relevant doses of the tracer (215 μCi/mouse) and of the MCT1 inhibitor (5 mg/Kg). PET/CT scans further revealed that [^18^F]-FLac primarily clears through urine.

^18^F-fluorine in [^18^F]-FLac has a relatively long half-life (110 min) compared to other potential radiolabels such as ^11^C (half-life = 20 min) or other potential tracers such as hyperpolarized ^13^C lactate (relaxation time in the order of 20 to 60 s). However, although it was a late event, potential defluorination occurred, as bone and joint labeling was observed 1 h after [^18^F]-FLac delivery. This effect was limited. If confirmed, defluorination would be consistent with a conversion of 3-fluoro-2-hydroxypropionate into 3-fluoropyruvate by LDH, followed by an *in vivo* defluorination of [^18^F]-3-fluoropyruvate [51], which could be facilitated by a nucleophilic attack of the keto group of [^18^F]-3-fluoropyruvate by thiol groups of proteins. Another possibility would be an active but slow uptake of [^18^F]-FLac by bone-associated cells.

Overall, our study offers the proof-of-principle that [^18^F]-FLac can be used as a PET tracer of lactate uptake. In oncology, [^18^F]-FLac could be used as a tool to predict and document a response to pharmacological agents and treatments aimed at disrupting lactate use and consumption by tumors, thus allowing to adapt treatment on an individual scale. Future studies will aim to generalize our findings to other tumor types and to investigate whether [^18^F]-FLac could also potentially be used as a diagnostic tool to evidence altered lactate metabolism in other pathologies than cancer, for example in cryptic exercise intolerance [52] and in epilepsy [4]. Because MCTs are stereoselective [12] for *L*- *vs*. *D*-lactate, the resolution of the enantiomers present in (±)-[^18^F]-3-fluoro-2-hydroxypropionate could further improve the selectivity of the tracer.

## MATERIALS AND METHODS

### Chemicals

[^18^O]-H_2_O was from Rotem. Benzyl acrylate was from Alpha Aesar; DMSO and tetrabutylammonium bicarbonate (TBAHCO_3_) from ABX; H_3_PO_4_ from Riedel-de Haën; Kryptofix 2.2.2. from Merck; NaH_2_PO_4_ and HPLC acetonitrile from VWR; and CDCl_3_ and TMS from Euristop. All other reagents were from Sigma-Aldrich.

### High-performance liquid chromatography (HPLC)

HPLC was performed on Gilson equipment (305 and 302 pumps) equipped with UV/VIS-151 and γ-ray NaI detectors connected in series and monitored by a GABI Star interface module (Raytest). Columns were for semi-preparative HPLC: Dionex Supelco Discovery C18, 5 μm, 250×10 mm; for analytical HPLC: MN, 150/4.6 Nucleosil 100-5 C18, 150 mm, ID: 4.6 mm and IonPac AS15, Dionex.

### Production of [^18^F]-fluoride

[^18^F]-fluoride was produced on a medical-isotope cyclotron (IBA Cyclone 18/9) using a [^18^O]-H_2_O liquid target. After irradiation, the target water was passed through a Chromafix 30-PS-HCO_3_ (Macherey-Nagel) or Accel Plus QMA Sep-Pak light cartridge (Waters) to trap the [^18^F]-fluoride.

### Synthesis of (±)-[^18^F]-2-fluoropropionate

(±)-[^18^F]-2-fluoropropionate was synthesized *via* combined procedures that were previously described [42, 53] on a home-made remote controlled module dedicated to nucleophilic fluorination and piloted by LabView. Briefly, an Accel Plus QMA carbonate Sep-Pak light cartridge (130 mg) loaded with [^18^F]-fluoride was eluted to a reaction vessel using a 0.1 mL aqueous solution of 3.5 mg K_2_CO_3_(25.0 μmol)/15 mg Kryptofix 2.2.2. (40.0 μmol) diluted in acetonitrile (0.9 mL). Anhydrous [^18^F]-fluoride was obtained by azeotropic distillation with acetonitrile at 95°C under a stream of helium. Methyl 2-bromopropionate (5 μL) dissolved in anhydrous acetonitrile (1 mL) was then added to the [^18^F]-fluoride. The vial was sealed and heated at 110°C for 10 min. After cooling, the reaction mixture was diluted by 3 mL of water and passed through a neutral alumina cartridge (Waters) to discard unreacted [^18^F]-fluoride. (±)-[^18^F]-methyl 2-fluoropropionate was isolated by semi-preparative HPLC (water/acetonitrile 60/40, 3 mL/min), diluted with 30 mL of water, and loaded on 2 conditioned Oasis HLB cartridges (Waters). Cartridges were rinsed with 15 mL of water and then loaded with 2 N NaOH. After 2 minutes, (±)-[^18^F]-2-fluoropropionate was eluted with 5 mL of water, and pH was set to 7.0 by the addition of NaH_2_PO_4_.

### Synthesis of benzyl oxirane-2-carboxylate

3-Chloroperoxybenzoic acid (14.04 g) was added to a solution of benzyl acrylate (23.04 mmol in 90 mL of dry dichloromethane [DCM]). The reaction mixture was heated under reflux and stirring for 7 days. DCM (100 mL) was then added to the solution, and washed twice with a saturated aqueous solution of sodium carbonate. The remaining DCM fraction was concentrated to 30 mL (rotavapor vacuum), and ethyl acetate (150 mL) was added. This solution was washed twice with a saturated aqueous solution of sodium carbonate, and the recombined organic layers were dried over sodium sulfate, filtered and concentrated to dryness under reduced pressure. The crude was finally purified by silica gel chromatography using cyclohexane/ethyl acetate (95/5, 100 mL; and 10/90, 800 mL), and remaining volatiles were removed under vacuum to yield the desired compound.

### Synthesis of (±)-[^18^F]-3-fluoro-2-hydroxypropionate

In a first method (Method I), a Chromafix 30-PS-HCO_3_ cartridge loaded with [^18^F]-fluoride was eluted in reverse order to a reaction vessel using a solution of 0.075 M of tetrabutylammonium bicarbonate (TBAHCO_3_, 80 μL, 6 μmol) in acetonitrile (0.9 mL). Anhydrous [^18^F]-fluoride was obtained by azeotropic distillation with acetonitrile at 95°C under a stream of helium. Benzyl oxirane-2-carboxylate (10 μL) dissolved in anhydrous DMSO (1 mL) was added to the [^18^F]-fluoride and was reacted for 10 minutes at 120°C. After cooling, the reaction mixture was diluted with 3.5 mL of water and passed through a neutral alumina cartridge (Waters) to discard unreacted [^18^F]-fluoride. The (±)-[^18^F]-3-fluoro-2-hydroxypropionate precursor was isolated by semi-preparative HPLC (20 mM NaH_2_PO_4_/CH_3_CN 70/30, 3 mL/min, retention time = 21 min), diluted with water (1.5 x vol), and loaded on a conditioned C18 cartridge (Waters). The cartridge was rinsed with 10 mL of water and then loaded with 0.5 N NaOH. After 5 minutes, (±)-[^18^F]-3-fluoro-2-hydroxypropionate was eluted with 1 mL of water, and pH set to 7.0 by the addition of H_3_PO_4_.

In a second method (Method II), a Chromafix 30-PS-HCO_3_ cartridge loaded with [^18^F]-fluoride was eluted in reverse order to a reaction vessel using a 30 μL aqueous solution of 0.55 mg K_2_C_2_O_4_(3.0 μmol)/2.25 mg Kryptofix 2.2.2. (6.0 μmol) diluted in 1 mL of “Trace Select” methanol. Anhydrous [^18^F]-fluoride was obtained by azeotropic distillation with acetonitrile at 95°C under a stream of helium. Benzyl oxirane-2-carboxylate (10 μL) dissolved in anhydrous 2-methyl 2-butanol (1 mL) was added to the [^18^F]-fluoride. The vial was sealed and heated at 105°C for 10 minutes. Solvent was then evaporated to dryness at 100°C under a stream of helium. After cooling, the reaction mixture was diluted by 4.5 mL of an acetonitrile/water 1/2 solution, and passed through a neutral alumina cartridge to discard unreacted [^18^F]-fluoride. (±)-[^18^F]-3-fluoro-2-hydroxypropionate was then prepared as in Method I.

### Synthesis of reference compound benzyl 3-fluoro-2-hydroxypropionate

Olah's reagent (hydrogen fluoride pyridine: pyridine ∼30 %, hydrogen fluoride ∼70 %, 3.4 mL) was added dropwise to a solution of benzyl oxirane-2-carboxylate (1.12 g) in dry DCM (7.5 mL) cooled to 0°C. After reaching room temperature, the mixture was stirred for 35 h. The biphasic solution was added on a silica suspension in DCM (100 mL), then filtered and washed with 50 mL of DCM. The desired benzyl-3-fluoro-2-hydroxypropionate was further purified over silica gel chromatography.

### Lactate dehydrogenase assay

A potential reduction of 3-fluoropyruvate to 3-fluoro-2-hydroxypropionate by lactate dehydrogenase (LDH) was measured *in vitro* using a previously reported protocol [54]. Nonradioactive 3-fluoropyruvate (14.6 mg) was dissolved in 4 mL of double distilled water containing 10 IU of rabbit muscle LDH (Sigma) and 5.3 IU of formate dehydrogenase (Sigma). The reaction was started by adding NADH to a final concentration of 0.2 mM and sodium formate to a final concentration of 40 mmol. The final volume was adjusted to 5 mL with double distilled water. The reaction was carried out at 37°C for 24 h under constant, gentle shaking at 120 rpm. Then, solution was spun through a 10 kDa filter to remove enzymes, and 3-fluoro-2-hydroxypropionate was detected by HPLC-MS using an Accela U(HPLC) equipped with a Luna Phenomenex 250*4.60 HPLC column and a ThermoScientific LTQ – ORBITRAP – XL fitted an electrospray ionization source working in negative mode.

### Cells and gene silencing

SiHa human cervix squamous cell carcinoma and SDQ9 human laryngeal squamous cell carcinoma cells were from ATCC. Cells were routinely cultured in DMEM (Thermo Fischer) containing glucose (4.5 g/L), Glutamax and 10% FBS. MCT1-deficient and control SiHa cells were produced as previously described [36], using the following vectors from Open Biosystems: TRCN0000038340 (shMCT1-1) and TRCN0000038339 (shMCT1-2). Control shRNA (shCTR) was Addgene plasmid 1864. SiHa-CRISPR-MCT1 cells were produced using Sigma LentiCRISPR (Target Site GTATAGTCATGATTGTTGGTGG), and transformed cells were selected using puromycin.

### Western blotting

Western blotting was performed as previously described [54]. Primary antibodies were rabbit polyclonals against MCT1 (Merck Millipore #AB3538P) and MCT4 [55]; and mouse monoclonals against Hsp90 (BD Bioscience #610419), CD147 (BD Bioscience #555961) and β-actin (Sigma #A5441).

### Oximetry

Basal oxygen consumption rates were determined on a Seahorse XF96 bioenergetic analyzer according to manufacturer's recommendations. Twenty thousand cells per well were plated 24-h before the experiment in complete DMEM containing 10% FBS. Medium was replaced by DMEM without glucose and glutamine supplemented with 10% dialyzed FBS, *L*-lactate (10 mM) and ±*D*-glucose (25 mM) 18-h before measurements. Data are normalized to cell numbers measured right before oximetry measurements.

### *In vitro* tracer uptake assay

We used a modified version of the ^14^C-Lactate uptake assay described by Draoui *et al*. [25]. Briefly, 250,000 cells were plated in flat-bottom 24 well plates (t = 0). When cells were attached (t = 6 h), medium was replaced by DMEM without glucose and glutamine, containing 10% dialyzed FBS and 10 mM of *L*-lactate, pH 7.0. Cells were then incubated overnight at 37°C, 5% CO_2_. On the day of experiment (t = 24 h), cell medium was replaced by media containing AR-C155858 (10 μM), AZD3965 (10 μM) or vehicle, and the cells were incubated at 37°C for 1 h. At the end of incubation, media were removed and cells were briefly washed twice with a modified KREBS solution without glucose (HEPES 25mM, NaCl 120 mM, KCl 4.8 mM, KH_2_PO_4_ 1.2 mM, MgSO_4_ 1.2 mM, CaCl_2_ 2 mM). The solution was replaced by a KREBS solution containing 10 mM of *L*-Lactate, pharmacological agents at the same concentration as during pretreatment or vehicle, (±)-[^18^F]-2-fluoropropionate (45 μCi/mL) or [^18^F]-3-fluoro-2-hydroxypropionate (45 μCi/mL). Cells were incubated for 6 min, after which the solution was removed and the cells were washed 3 times with an ice-cold KREBS solution containing *L*-Lactate (10 mM). Cells were lysed with NaOH 0.1 N, and ^18^F activity was measured in the cell lysate using a Wiper Gold γ-counter (Laboratory Technologies). Activity is expressed as % of initial dose. For background determination, wells without cells were treated in the exact same way.

### *In vivo* tracer uptake assay

All *in vivo* experiments were performed with approval of UCL *Comité d’Ethique pour l’Expérimentation Animale* (approval ID 2014/UCL/MD/014) according to national and European animal care regulations. To avoid inter-subject variability, 500,000 SiHa-shCTR and SiHa-shMCT1 cells in a HBSS:Matrigel 1:1 solution were respectively injected in the left and right flank of same 6.5 week-old male NMRI nude mice. In experiments with SQD9 cells, 1,000,000 cells in HBSS:Matrigel 2:1 were injected in one flank of NMRI nude mice. Experiments were performed on tumors of ∼10 mm in diameter, *i.e*., 3-5 weeks after cancer cell inoculation. For intravenous injection, MCT1 inhibitor AR-C155858 (Tocris) was dissolved in 0.9% NaCl with 10% (2-hydroxypropyl)-β-cyclodextrin at a concentration of 2.5 mg/mL [56], and AZD3965 in 0.9% NaCl with 2.5% ethanol and 10% (2-hydroxypropyl)-β-cyclodextrin at a concentration of 2.5 mg/mL. (±)-[^18^F]-3-fluoro-2-hydroxypropionate (150-250 μCi) was injected in the tail vein of the animals 10 min after the delivery of AR-C155858 (5 mg/Kg) [56], AZD3965 (5 mg/Kg) or vehicle (70 μL). At indicated times, a whole body 10 min static PET imaging (small-animal Mosaic PET Scan system, Philips Medical Systems) directly followed by a 10 min transmission CT scan (NanoSPECT/CT Small Animal Imager, Bioscan; source: 370 MBq ^137^Cs; X-Ray tube voltage: 55kVp; number of projections: 180; exposure time 1,000 ms) were performed on isoflurane-anesthetized mice kept at 35°C. PET images were corrected for attenuation and reconstructed using fully 3D iterative algorithm 3D-RAMLA in a 128 × 128 × 120 matrix, with a voxel size of 1 mm^3^. CT images were reconstructed with a voxel size of 0.221 × 0.221 × 0.221 mm^3^. 2D Regions of interest (ROIs) were manually delineated on PET images using the PMOD software version 3.5 (PMOD technologies Ltd). Tumor localization was determined on PET/CT fused images. Ribcage and skin were used as internal and external limits, respectively. Tracer uptake is expressed as standard uptake value (SUV) calculated on the mean value of voxels within the manually defined 3D volume of interest (VOI). Manually defined VOIs were used to measure tumor volumes.

### Statistics

Data were analyzed using GraphPad Prism version 6.04 for Windows. All results are expressed as mean ± SEM. *N* refers to the number of independent experiments and *n* to the total number of replicates per treatment condition. Error bars are sometimes smaller than symbols. Student's *t* test and one-way ANOVA were used where appropriate. *P* < 0.05 was considered to be statistically significant.

## SUPPLEMENTARY MATERIALS FIGURES AND TABLES














