# Supplementary material for: Radiosynthesis and validation of (±)-[18F]-3-fluoro-2-hydroxypropionate ([18F]-FLac) as a PET tracer of lactate to monitor MCT1-dependent lactate uptake in tumors
Source: Oncotarget. 2017 Jan 17;8(15):24415–28. doi: 10.18632/oncotarget.14705 (PMC5421858; doi:10.18632/oncotarget.14705)
Supplement: Supplementary file 1 [file oncotarget-08-24415-s001.pdf]

Radiosynthesis and validation of (±)-[^{18}F]-3-fluoro-2-hydroxypropionate ([^{18}F]-FLac) as a PET tracer of lactate to monitor MCT1-dependent lactate uptake in tumors

SUPPLEMENTARY FIGURES AND VIDEOS

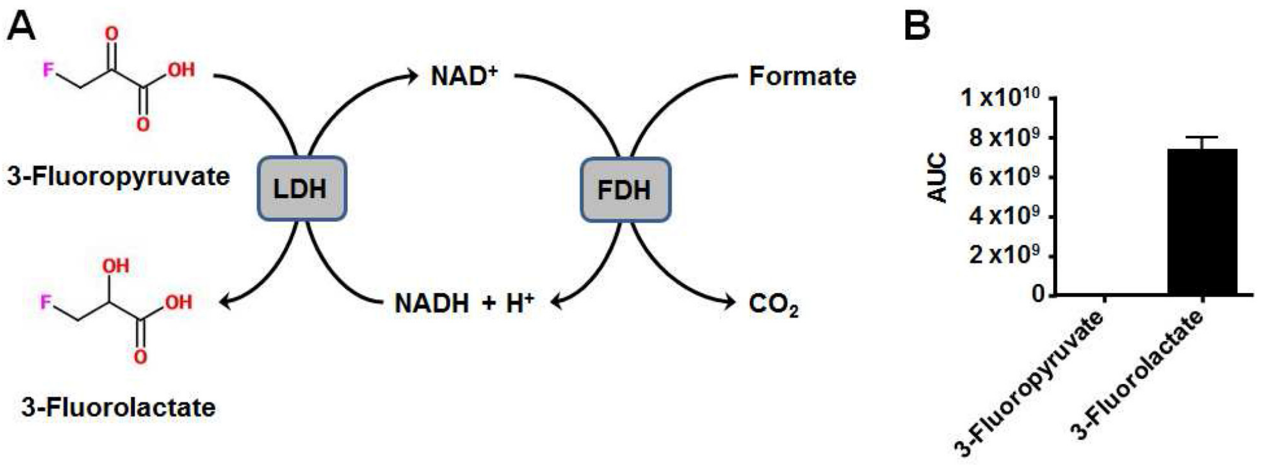

Supplementary Figure 1: 3-fluoropyruvate can be reduced to 3-fluorolactate by lactate dehydrogenase (LDH). A. Scheme of the reaction used for 3-fluoropyruvate reduction. **B.** 3-fluoropyruvate and 3-fluorolactate detection using mass spectrometry after the reaction schematized in A (N = 1, n = 3).

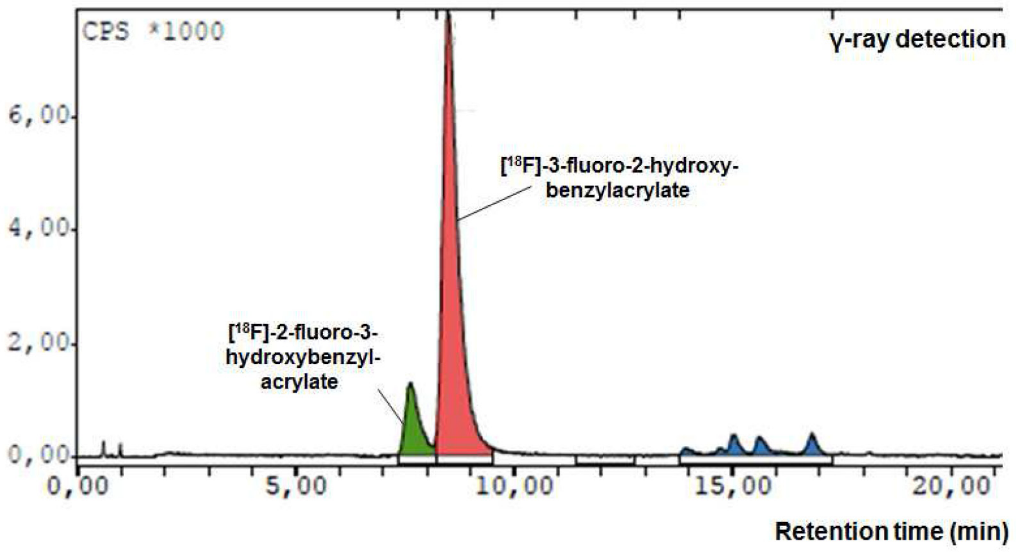

Supplementary Figure 2: Elution spectrum of (±)-[^{18}F]-benzyl 3-fluoro-2-hydroxypropionate and (±)-[^{18}F]-2-fluoro-3-hydroxybenzylacrylate on a Supelco Discovery C18 HPLC column equipped with a NaI γ-ray detector, showing regioisomer ratio when 2-methyl 2-butanol was used as a fluorination solvent (Method II).

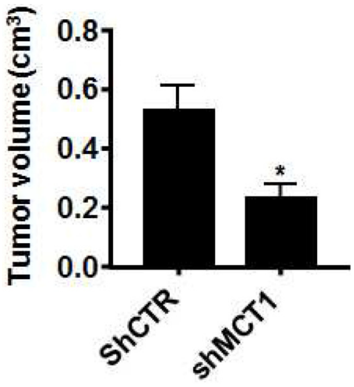

Supplementary Figure 3: MCT1 silencing induces a growth retardation of SiHa tumors in mice. Mice were bearing 2 SiHa tumors expressing a control shRNA (shCTR) in one flank or a shRNA against MCT1 (shMCT1) in the other flank. Tumor volumes were measured on CT images obtained 36 days after tumor implantation (* $p < 0.05$; N = 2, n = 6-7).

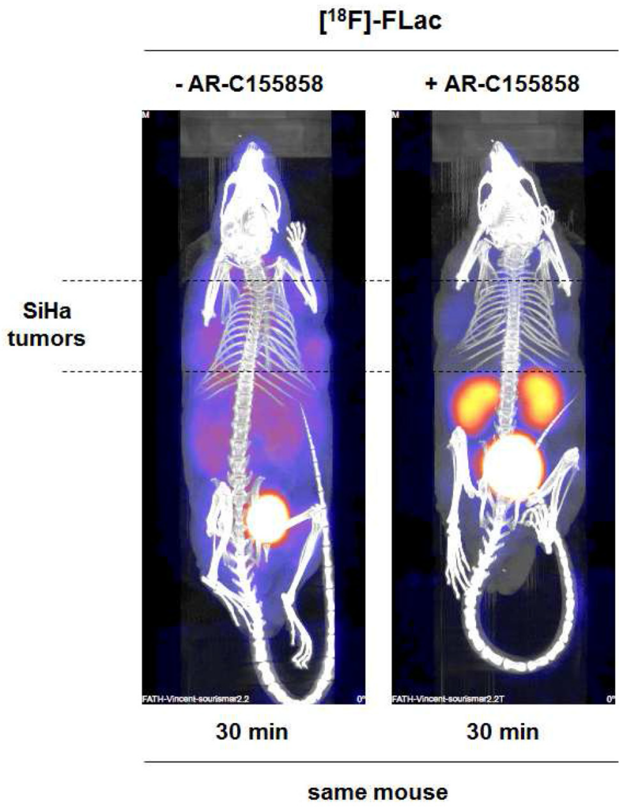

Supplementary Video 1: (±)-[^{18}F]-3-fluoro-2-hydroxypropionate ([^{18}F]-FLac) allows to predict and to document a tumor response to MCT1 inhibition by AR-C155858 in human SiHa cervix tumors in mice. Animated pictures are available online. The movie shows PET/CT scans of a mouse bearing 2 SiHa tumors. A same mouse received an intravenous injection of (±)-[^{18}F]-3-fluoro-2-hydroxypropionate ([^{18}F]-FLac; 200-250 μCi) on 2 consecutive days before (left movie) and after (right movie) a bolus delivery of AR-C155858 (5 mg/Kg) intravenously 10 min before tracer injection. The mouse was imaged 30 min after [^{18}F]-FLac injection. Color scale is normalized for the injected dose and the animal weight.

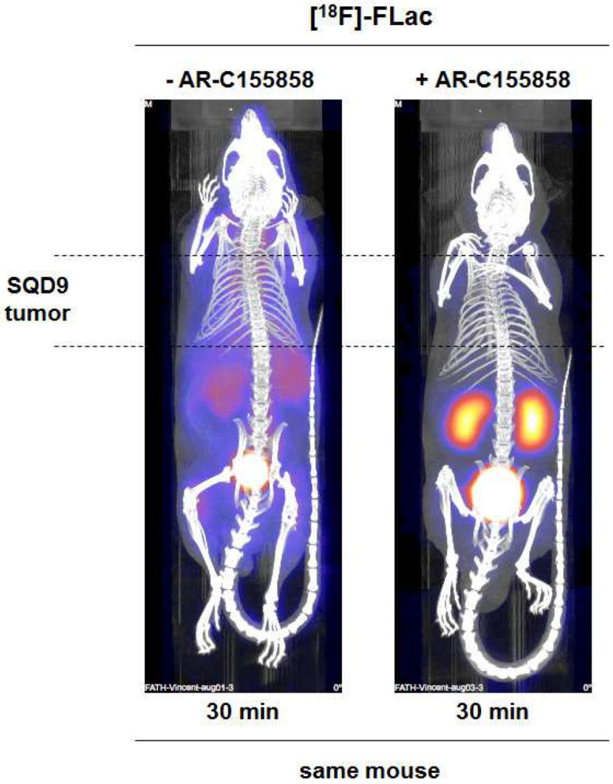

Supplementary Video 2: (±)-[^{18}F]-3-fluoro-2-hydroxypropionate ([^{18}F]-FLac) allows to predict and to document a tumor response to MCT1 inhibition by AR-C155858 in human SQD9 head and neck tumors in mice. Animated pictures are available online. The movie shows PET/CT scans of a mouse bearing a SQD9 tumor. A same mouse received an intravenous injection of (±)-[^{18}F]-3-fluoro-2-hydroxypropionate ([^{18}F]-FLac; 200-250 µCi) on 2 consecutive days before (left movie) and after (right movie) a bolus delivery of AR-C155858 (5 mg/Kg) intravenously 10 min before tracer injection. The mouse was imaged 30 min after [^{18}F]-FLac injection. Color scale is normalized for the injected dose and the animal weight.

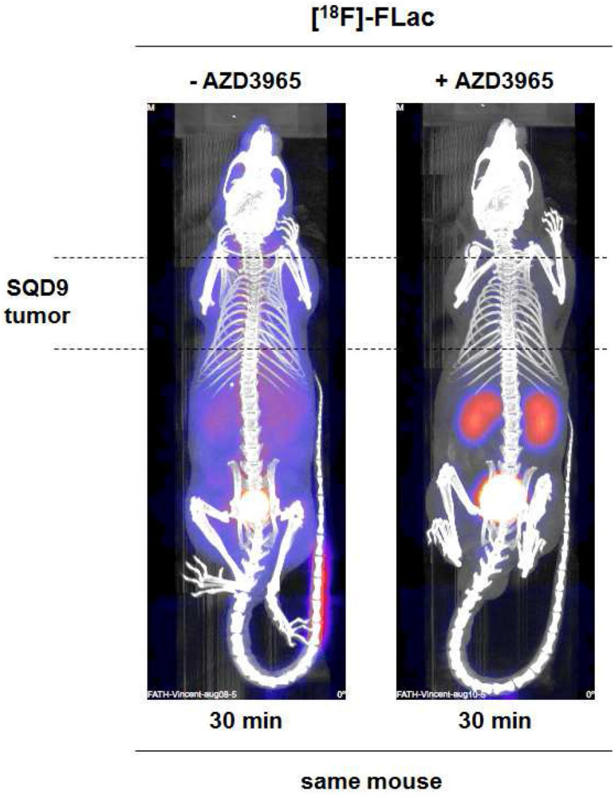

Supplementary Video 3: (±)-[^{18}F]-3-fluoro-2-hydroxypropionate ([^{18}F]-FLac) allows to predict and to document a tumor response to MCT1 inhibition by AZD3965 in human SQD9 head and neck tumors in mice. Animated pictures are available online. The movie shows PET/CT scans of a mouse bearing a SQD9 tumor. A same mouse received an intravenous injection of (±)-[^{18}F]-3-fluoro-2-hydroxypropionate ([^{18}F]-FLac; 200-250 µCi) on 2 consecutive days before (left movie) and after (right movie) a bolus delivery of AZD3965 (5 mg/Kg) intravenously 10 min before tracer injection. The mouse was imaged 30 min after [^{18}F]-FLac injection. Color scale is normalized for the injected dose and the animal weight.
