# Supplementary figures and images for: Radiosynthesis and validation of (±)-[18F]-3-fluoro-2-hydroxypropionate ([18F]-FLac) as a PET tracer of lactate to monitor MCT1-dependent lactate uptake in tumors
Source: Oncotarget. 2017 Jan 17;8(15):24415–28. doi: 10.18632/oncotarget.14705 (PMC5421858; doi:10.18632/oncotarget.14705)

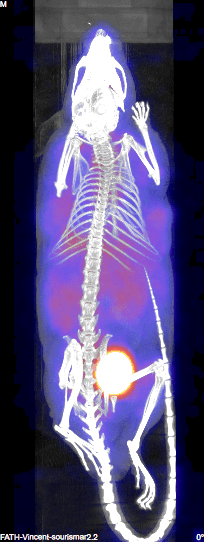

Supplement: Supplementary file 2 [file oncotarget-08-24415-s002.gif]

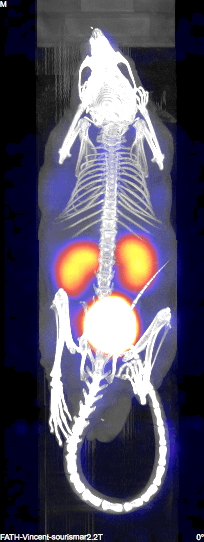

Supplement: Supplementary file 3 [file oncotarget-08-24415-s003.gif]

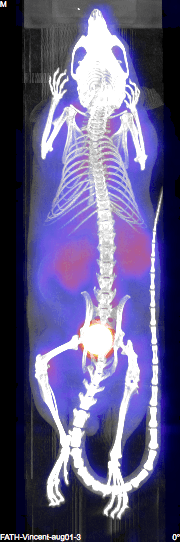

Supplement: Supplementary file 4 [file oncotarget-08-24415-s004.gif]

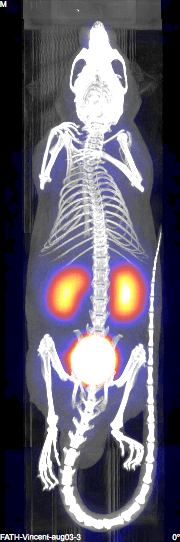

Supplement: Supplementary file 5 [file oncotarget-08-24415-s005.gif]

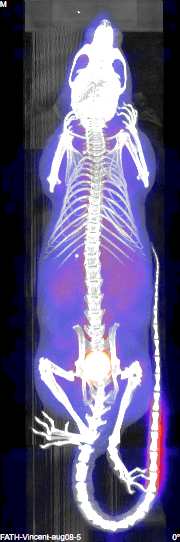

Supplement: Supplementary file 6 [file oncotarget-08-24415-s006.gif]

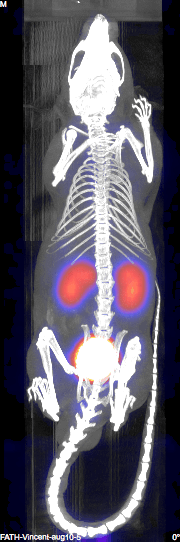

Supplement: Supplementary file 7 [file oncotarget-08-24415-s007.gif]
